# Color changes of three different brands of acrylic teeth in removable dentures in three different beverages: An in vitro study

**DOI:** 10.34172/joddd.2020.034

**Published:** 2020-09-21

**Authors:** Roza Motayagheni, Zia Ebrahim Adhami, Seyede Mahsa Taghizadeh Motlagh, Fereshte Mehrara, Neda Yasamineh

**Affiliations:** ^1^Department of Prosthodontics, Faculty of Dentistry, Tabriz University of Medical Sciences, Tabriz, Iran; ^2^Department of Pediatrics, Faculty of Dentistry, Tabriz University of Medical Sciences, Tabriz,Iran; ^3^DDS. Tabriz University of Medical Sciences, Tabriz, Iran; ^4^Dental and Periodontal Research Center, Tabriz University of Medical Sciences, Tabriz, Iran

**Keywords:** Acrylic resin teeth, Color change, Ivoclar BetaDent, BStar

## Abstract

**Background.** The best prosthetic appliances exhibit a high level of similarity to the lost organ. Color should exhibit favorable stability, as a critical factor in the esthetic appearance of dental prostheses and materials used in fabricating prostheses. The present in vitro study was undertaken to evaluate color changes of three different brands of acrylic resin teeth in three different beverages.

**Methods.** In this in vitro study, 10 samples from each brand of acrylic resin tooth (Ivoclar, Italy; BStar, Iran; and BetaDent, Iran) were immersed in the following beverages for one week: tea, orange juice, natural carrot juice, and distilled water (control). The color parameters were measured using the spectrophotometry technique before and after immersion, and changes were calculated. The same procedures were carried out with the same number of samples at 30-day interval. Data were analyzed with multivariate ANOVA and post hoc Tukey tests.

**Results.** After seven days, Ivoclar and BStar brands exhibited the minimum (1.78) and maximum (3.39) color changes, respectively (P<0.05). At the 30-day interval, the Ivoclar and BetaDent brands exhibited the minimum (3.03) and maximum (4.27) color changes, respectively (P<0.05). At the 7-day interval, carrot juice, orange juice and tea, in descending order, caused the maximum and minimum color changes. At the 30-day interval, carrot juice resulted in maximum color changes (P<0.05); orange juice, and tea caused similar color changes (P>0.05).

**Conclusion.** Different beverages resulted in color changes with different patterns in different brands of acrylic resin teeth. Overall, the Ivoclar brand exhibited less color changes compared to BStar and BetaDent brands. In the first week, all the three brands and in 30 days, Ivoclar and BStar brands exhibited clinically acceptable color changes.

## Introduction

Complete dentures are fabricated for the functional reconstruction of natural teeth. An ideal arrangement of acrylic resin teeth can provide maximum stability, comfort, esthetics, and function for the patients.^1-3^ Undoubtedly, the best prostheses exhibit the highest resemblance to the lost organ. Color is an essential parameter that affects the esthetics of dental prostheses; therefore, the materials used to fabricate prostheses should exhibit very high color stability because they continually become exposed to various foods and drinks in the oral cavity. Color changes might occur as a result of internal or external factors. The internal factors mainly exert their effect by changing the chemical structure of the materials; however, external factors depend on the surface characteristics of the material and the oral cavity environment.^4-6^

Of all the different materials used for the fabrication of complete dentures, acrylic resin teeth are the most commonly used because they can create chemical bonds with denture base acrylic rein, are light in weight, are less prone to fracture and are easily adjusted for proper occlusion.^5,7,8^ However, these teeth exhibit less resistance to abrasion and have low color stability.^5,9-11^

Several studies have evaluated and compared the color stability of artificial teeth from different brands. The majority of these studies have shown that different artificial tooth banks have different stability.^6,8^ Mousavi et al^6^ carried out an in vitro study to compare color changes of acrylic resin teeth from three different brands (Apple, Iran; Ivoclar, Italy; and PolyDent, Slovenia) in three beverages (tea, a soft drink, and coffee) using the spectrophotometry technique at 1-, 3-, and 6-week intervals. The results showed that Apple and Ivoclar brands exhibited the highest and lowest color changes, respectively. In addition, each beverage resulted in a different color change pattern in each brand. In a study by Mutlu-Sagesen et al,^11^ the color stability of porcelain teeth was higher than that of acrylic resin teeth.

Hipólito et al^8^ compared color changes of 10 different brands of acrylic resin teeth in a soft drink, coffee, and orange juice at 1-, 7-, 15-, and 30-day intervals using spectrophotometry technique. The results showed different color change patterns in different acrylic resin brands in each beverage. In that study, the soft drink and coffee caused more color changes compared to orange juice and saliva.

Ansari et al^12^ compared the color stability of Glamour composite resin and Ideal Dent acrylic resin teeth in tea and coffee and reported that Glamour composite rein teeth underwent more color changes than Ideal Dent acrylic resin teeth; however, both color changes were in the acceptable range clinically. Shaegh and Bagherani^13^ reported that the color change of Ideal Maku acrylic rein teeth was similar to that of the Ivoclar acrylic resin teeth. In the study above, visual evaluations were used to compare color changes between the samples.

Koksal et al^5^ compared the color stability of two porcelain tooth brands and three brands of acrylic resin teeth in vitro. The samples were immersed in coffee, tea, and a soft drink and in distilled water as a control. The following results were reported:

1) The porcelain artificial teeth exhibited more color changes compared to acrylic resin artificial teeth;

2) The color changes increased with an increase in the duration of immersion;

3) The most chromogenic beverage was instant coffee.

Gregorius et al^14^ studied the effect of the aging process and staining on acrylic teeth and concluded that all the teeth immersed in the three solutions that underwent an aging process exhibited excellent color stability.

Roslan et al^15^ examined the color changes of acrylic teeth in turmeric and black coffee solutions and reported that the artificial teeth exhibited more color changes in turmeric solution than black coffee.

Waldemarin et al^16^ examined the color changes of dentures using different processing techniques. Artificial teeth were immersed in distilled water, cola, coffee, red wine, and yerba mate tea. The results showed that professionals could identify the color change of teeth in yerba mate tea, but color changes in red wine could be discerned by patients and were not clinically acceptable.

In recent years, dental equipment manufacturers have marketed different types of artificial teeth; therefore, it is necessary to evaluate the quality of these teeth and collect evidence-based data so that the best choice can be made among these types and brands. The present in vitro study evaluated and compared the color changes of three different brands of acrylic resin teeth (Ivoclar, Italy; BStar, Iran, and BetaDent, Iran) in three different beverages.

## Methods

Eighty acrylic resin teeth from each brand (Vivadent, Ivoclar, Italy; BetaDent, Iran; and BStar, Iran) were included in this in vitro study (a total of 240 samples).

The sample size was determined using the results of a study by Mousavi et al^6^ and by considering a color change of 3.3±0.02 in tea and a difference of 10%, α=0.05, and a study power of 80%. In this context, 10 samples were assigned to each subgroup, amounting to 240 samples.

**Figure 1:**
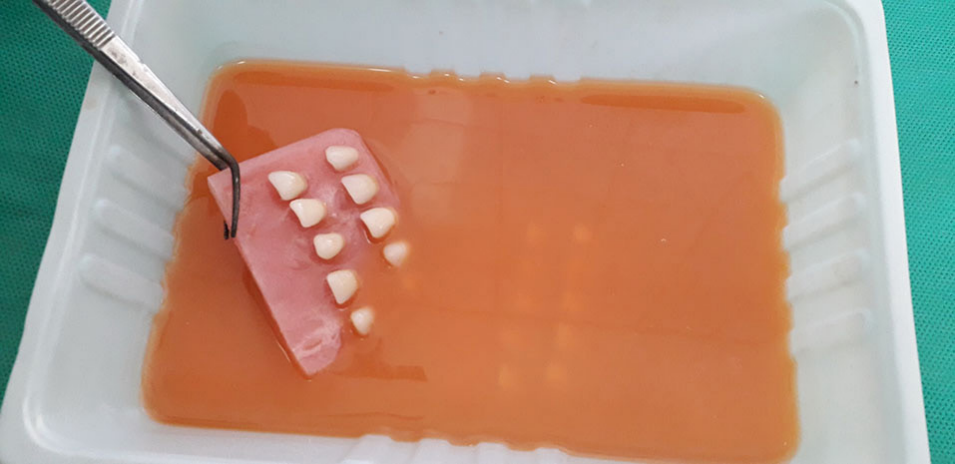
Samples immersed in beverages.

**Figure 2:**
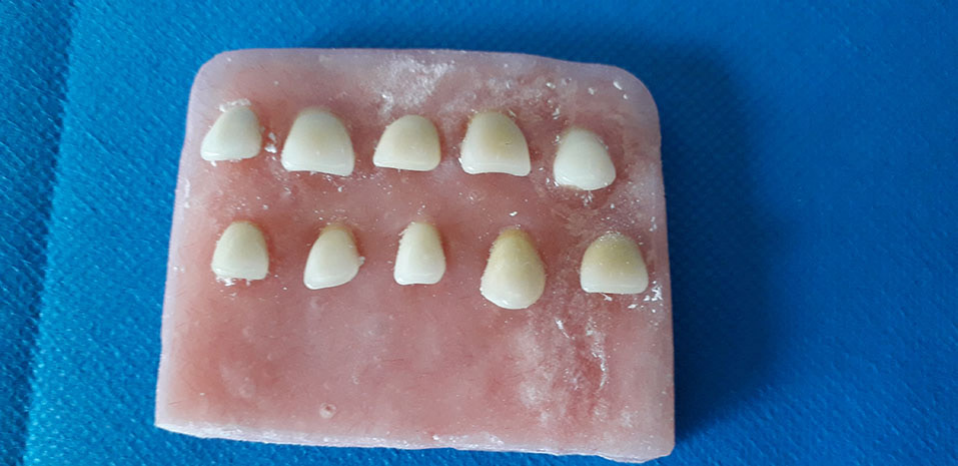
Samples in heat-cured acrylic resin base.

**Figure 3:**
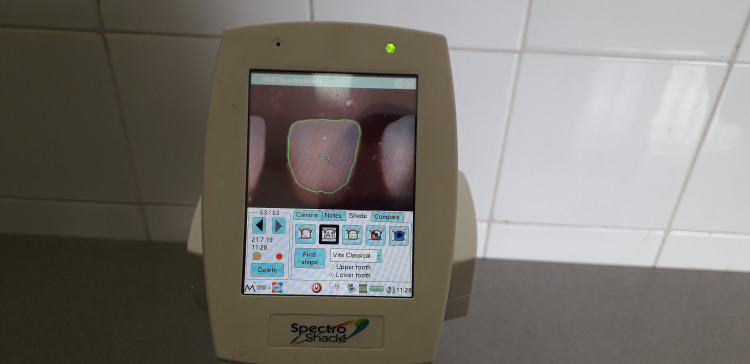
A spectrophotometer was used to determine color parameters.

Ten samples from each acrylic resin brand were immersed in tea, orange juice, natural carrot juice, and in distilled water (as a control) for one week (Figure 1). The teeth had no contact with each other in the solutions. Before immersion, the teeth underwent heat-curing procedures in an acrylic resin base to simulate removable denture fabrication procedures (Figure 2). A spectrophotometer (SpectroShade Micro II, SpectroShade USA) was used to determine color parameters before and after immersion, and the overall changes in the color parameters were calculated (Figure 3). The procedures were repeated with a similar number of samples after 30 days. Separate samples were used for 7- and 30-day intervals.

The following formula was used to determine the color changes of each sample in each beverage for a, b, and L values at baseline before immersion and at 7- and 30-day intervals after immersion:

ΔE=√(Δa)^2^+(Δb)^2^+(ΔI)^2^

where Δa, Δb, and Δl show the differences before and after immersion.

The data were reported using descriptive statistical techniques (mean ± SD). Multivariate ANOVA and post hoc Tukey tests were used to compare color changes after immersion in the three beverages and in distilled water between the three acrylic resin brands at T_0_=0, T_1_=7 days, and T_2_=30 days. Shapiro-Wilk test was used to assess the normality of data. SPSS 17 was used for statistical analyses. Statistical significance was defined at P<0.05.

## Results

Shapiro-Wilk test was used to evaluate data normality, which showed that each combination of data (a combination of tooth brand and the beverage) was normally distributed.

### Evaluation of color changes in the Ivoclar brand

**Figure 4:**
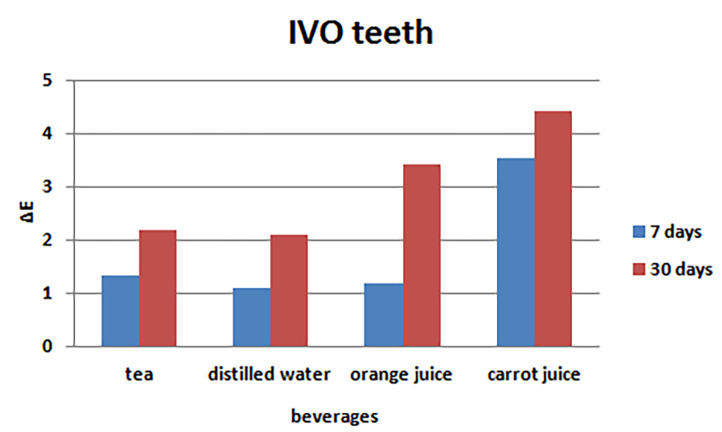
The mean color changes 7 and 30 days after immersion in terms of tooth brand.

One-way ANOVA revealed significant differences in the mean color changes of the Ivoclar brand at 7- and 30-day intervals between tea, distilled water, orange juice, and carrot juice (P<0.001), with the minimum ΔE in distilled water (control) and the maximum ΔE in carrot juice at both 7- and 30-day intervals (Table 1). Post hoc Tukey tests were used for two-by-two comparisons. (Figure 4) presents the bar graphs of the mean color changes 7 and 30 days after immersion in the beverages under study.

**Table 1:** Comparison of mean color changes in the Ivoclar artificial teeth after immersion in the study beverages

**Tooth brand**	**Time**	**Beverage**	**No.**	**Mean**	**SD**	**P-value**
**Ivoclar**	**7 days**	**Tea**	10	1.34	0.76	<0.001
		**Distilled water**	10	1.11	0.27	
		**Orange juice**	10	1.17	0.33	
		**Carrot juice**	10	3.52	0.95	
	**30 days**	**Tea**	10	2.18	0.38	<0.001
		**Distilled water**	10	2.1	0.25	
		**Orange juice**	10	3.41	1.25	
		**Carrot juice**	10	4.43	1.07	

### Evaluation of color changes in the BStar brand

**Figure 5:**
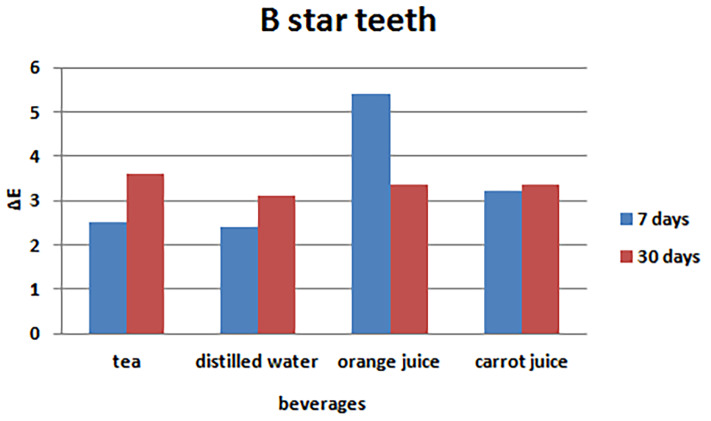
The mean color changes of BStar artificial teeth after immersion in the study beverages for 7 and 30 days.

The results of one-way ANOVA showed significant differences in the mean color changes of BStar artificial teeth after seven days of immersion in tea, distilled water, orange juice, and carrot juice (P=0.001), with the minimum ΔE in distilled water (control) and maximum ΔE in orange juice. In addition, based on one-way ANOVA results, there were no significant differences in color changes 30 days after immersion in the beverages (P=0.734) (Table 2). Post hoc Tukey tests were used for two-by-two comparisons after seven days. Two-by-two comparisons were not made after 30 days because ANOVA did not reveal any significant differences (Table 3). Figure 5 presents the bar graph of mean color changes 7 and 30 days after immersion in the beverages.

**Table 2:** Comparison of the mean color changes in the study beverages in BStar artificial teeth

**Tooth brand**	**Time**	**Beverage**	**No.**	**Mean**	**SD**	**P-value**
**BStar**	**7 days**	**Tea**	10	2.51	1.38	0.001
		**Distilled water**	10	2.40	0.95	
		**Orange juice**	10	5.42	2.47	
		**Carrot juice**	10	3.23	0.82	
	**30 days**	**Tea**	10	3.60	0.54	0.734
		**Distilled water**	10	3.10	1.13	
		**Orange juice**	10	3.37	1.49	
		**Carrot juice**	10	3.35	1.12	

**Table 3:** Two-by-two comparisons with the use of Tukey tests

**Tooth brand**	**Time**	**Beverage**		**Mean difference ( i -j)**	**SD error**	**P-value**
		**I**	**j**			
**BStar**	**7 days**	**Tea**	**Distilled water**	0.11	0.70	0.838
			**Orange juice**	-2.91	0.70	0.001
			**Carrot juice**	-0.72	0.70	0.178
		**Distilled water**	**Orange juice**	-3.02	0.70	0.001
			**Carrot juice**	-0.83	0.70	0.268
		**Orange juice**	**Carrot juice**	2.19	0.70	0.017

### Evaluation of color changes of BetaDent brand

**Figure 6:**
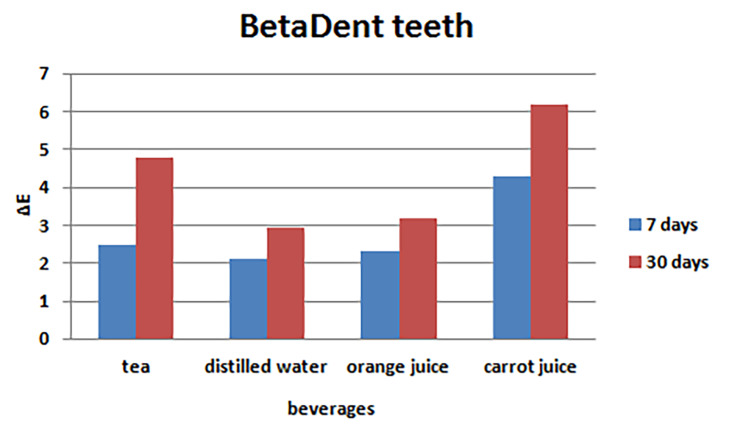
The mean color changes of BetaDent artificial teeth 7 and 30 days after immersion in the study beverages.

The results of one-way ANOVA showed significant differences in the mean color changes of BetaDent artificial teeth 7 and 30 days after immersion in tea, distilled water, orange juice, and carrot juice (P<0.001), with minimum ΔE in distilled water and maximum ΔE in carrot juice both 7 and 30 days after immersion (Table 4). Figure 6 presents the bar graph of color changes 7 and 30 days after immersion in the beverages.

**Table 4:** Comparison of the means of color changes of BetaDent artificial teeth after immersion in the study beverages

**Tooth brand**	**Time**	**Beverage**	**No.**	**Mean**	**SD**	**P-value**
**BetaDent**	**7 days**	**Tea**	10	2.50	0.40	<0.001
		**Distilled water**	10	2.10	0.04	
		**Orange juice**	10	2.30	0.38	
		**Carrot juice**	10	4.31	1.33	
	**30 days**	**Tea**	10	4.80	0.66	<0.001
		**Distilled water**	10	2.95	0.27	
		**Orange juice**	10	3.18	1.03	
		**Carrot juice**	10	6.17	0.85	

### Evaluation of the effect of a combination of beverages and tooth brand on color changes of the teeth 7 and 30 days after immersion in the beverages

### Evaluation of the effect of a combination of tooth brand and beverage type seven days after immersion

Two-way ANOVA was used to compare the cumulative effect of tooth type and solution type on color changes. The results showed that seven days after immersion, such an effect on color change was significant (P<0.001) (Table 5).

**Table 5:** The means of color changes in terms of the tooth brand and solution types after 7 days of immersion

		**Mean**	**SD error**	**P-value** ^*^
**Solution**	**Tea**	2.115	0.252	<0.001
	**Distilled water**	1.870	0.252	
	**Orange juice**	2.967	0.252	
	**Carrot juice**	3.690	0.252	
**Tooth brand**	**Ivoclar**	1.785	0.218	<0.001
	**BStar**	3.390	0.218	
	**BetaDent**	2.804	0.218	

P-values from two-way ANOVA

**Figure 7:**
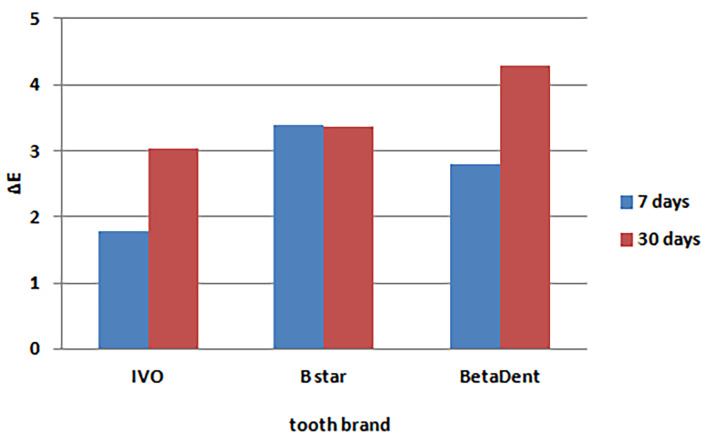
The mean color changes 7 and 30 days after immersion in terms of tooth brand.

**Figure 8:**
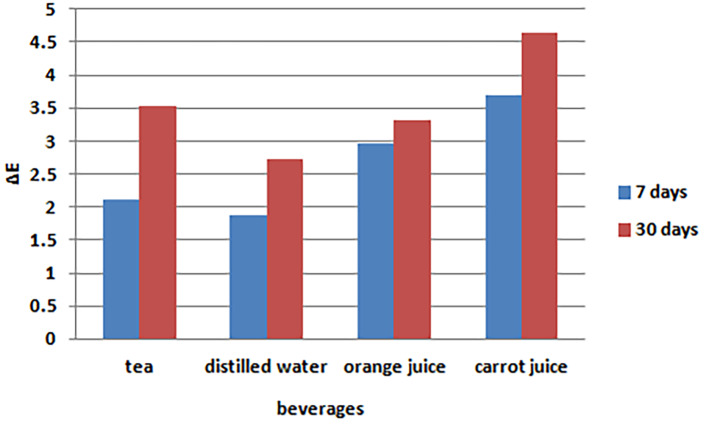
The mean color changes 7 and 30 days after immersion in terms of beverages.

Based on the data presented in Table 5, the minimum mean color changes occurred in distilled water (control), and the maximum mean color changes occurred in carrot juice. In addition, the minimum changes were detected in the Ivoclar brand, and the maximum changes were detected in the BStar brand. Figures 7 and 8 present the bar graphs of the mean color changes in terms of the tooth brand and beverage type.

### Evaluation of the cumulative effect of tooth brand and beverage type on color changes 30 days after immersion

Two-way ANOVA was used to compare the cumulative effects of tooth brand and beverage type on color changes. The tooth brand and beverage type had significant effects on color changes in teeth three days after immersion based on the results of two-way ANOVA (P<0.001) (Table 6). The minimum and maximum mean color changes were detected in distilled water (control) and carrot juice, respectively. In addition, the Ivoclar and BetaDent artificial teeth exhibited the minimum and maximum color changes, respectively.

**Table 6:** The mean color changes after 30 days of immersion in terms of tooth brand and beverage type

		**Mean**	**SD error**	**P-value** ^*^
**Solution**	**Tea**	3.526	0.226	<0.001
	**Distilled water**	2.716	0.226	
	**Orange juice**	3.323	0.226	
	**Carrot juice**	4.652	0.226	
**Tooth brand**	**Ivoclar**	3.030	0.196	0.006
	**BStar**	3.355	0.196	
	**BetaDent**	4.277	0.196	

P-values of two-way ANOVA

## Discussion

Color stability of artificial teeth is a critical factor in the esthetics of dental prostheses. Artificial teeth are continuously exposed to various foods and beverages; therefore, they should have proper color stability. Color changes in dental materials are evaluated with the use of clinical methods or a spectrophotometer. The use of a spectrophotometer improves the accuracy of measurements and decreases human error.^4,6^ In the present study, the color changes of three different brands of artificial teeth were evaluated after immersion in three different beverages, with the use of a spectrophotometer (SpectroShade Micro II – SpectroShade USA) and the CIELab technique.

The results showed that at the 7-day interval, Ivoclar, BetaDent, and BStar brands exhibited the lowest and highest color changes in ascending order. At the 30-day interval, too, Ivoclar artificial teeth exhibited the minimum color changes. The BStar artificial teeth exhibited color changes comparable to the Ivoclar brand and less than the BetaDent brand.

Previous studies have reported that color changes of <3.3 are clinically acceptable.^3^ According to the present study, seven days after immersion, all the three brands of acrylic rein teeth, and at the 30-day interval, Ivoclar and BStar brands exhibited clinically acceptable color changes.

Mousavi et al^6^ carried out an in vitro study and evaluated and compared color changes of three different brands of acrylic resin teeth (Apple, Iran; Ivoclar, Italy; and PolyDent, Slovenia) in tea, a soft drink, and coffee with the use of a spectrophotometer at 1-, 3-, and 6-week intervals. The results showed almost similar color changes in all the three brands in tea at 1-, 3-, and 6-week intervals, which were acceptable clinically (a color change of <3.3), consistent with the results of the present study, in which all the brands exhibited clinically acceptable color changes in all the three brands in tea during the first seven days.

In the study by Mousavi et al,^6^ the mean color change of the Ivoclar brand at 1-, 3-, and 6-week intervals was approximately 3.4, which was higher than that of this brand in the present study (mean color changes of 1.34 on the 7th day and 2.18 on the 30th day for Ivoclar). The Iranian brand, Apple, in the study by Mousavi et al^4^ exhibited greater color changes one week after immersion in tea (3.1) compared to the Iranian brands of BStar (2.51) and BetaDent (2.50) in the present study. Overall, Mousavi et al^4^ concluded that color changes of the Iranian brand, Apple, at all the three study intervals and in the three beverages (tea, coffee, and a soft drink) were greater than those of the Ivoclar brand and clinically unacceptable. However, in the present study, only the BetaDent brand exhibited color changes greater than the clinically acceptable range at the 30-day interval.

Overall, in the present study, at the 7-day interval, carrot juice, orange juice, and tea resulted in the maximum and minimum color changes, respectively. In addition, at 7- and 30-day intervals, the carrot juice induced the greatest color changes in the Ivoclar and BetaDent brands. In the BStar brand, the orange juice induced the greatest color changes at the 7-day interval; however, at the 30-day interval, although tea induced the greatest color change, the difference was not significant statistically. In the study by Mousavi et al,^4^ different solutions induced different color change patterns in each brand, consistent with the present study.

In a study by Hipólito et al,^8^ the spectrophotometric technique was used to evaluate color changes of 10 different brands of acrylic resin teeth in a soft drink, coffee, and orange juice at 1-, 7-, 15-, and 30-day intervals. Based on the results, the different brands of acrylic resin teeth exhibited different color change patterns in each solution, consistent with the results of the present study. In the study above, the soft drink and coffee induced greater color changes compared to orange juice and saliva. In addition, orange juice induced less color changes at 7-day (0.72–1.71) and 30-day (0.72–2.79) intervals compared to the present study (3.32–2.96). However, in both studies, the color changes were in the clinically acceptable range.

Ansari et al^12^ compared the color stability of Glamour composite resin teeth with Ideal Dent acrylic resin teeth in tea and coffee and reported that Glamour composite resin teeth exhibited greater color changes compared to Ideal Dent teeth. However, both color changes were clinically acceptable.

Shaegh and Bagherani^13^ reported that the color stability of Ideal Maku acrylic resin teeth was similar to that of the Ivoclar brand. It should be pointed out that they used visual evaluations to compare color changes of the samples.

Roslan et al^15^ studied the color changes of acrylic teeth in two solutions of turmeric and black coffee. Color changes were measured at 1-, 7-, and 15-day intervals. The color changes in turmeric solution were the highest, which increased over time. In the present study, the greatest color change was related to carrot juice, which has an orange color; similarly, the color change in acrylic teeth increased over time.

Gregorius et al^14^ evaluated the color stability of high-strength acrylic resin teeth. Four different colors from three different brands were evaluated. In the first phase, the teeth were immersed in distilled water, coffee, and red wine. In phase two, the acrylic teeth were subjected to an aging process by a weatherometer. In this study, different brands exhibited different color change patterns under the influence of different solutions similar to the present study, and the least color change in red wine was related to the Vita Physiodent brand.

Acrylic resin teeth are nufactured using polymethyl methacrylate with a high conversion rate and a high dibenzoyl peroxide content, which has been reported to be responsible for color changes of these teeth. The use of hydrophilic materials in the fabrication process of acrylic resin teeth is another important factor in the color change of these teeth. The use of hydrophobic materials, such as 2-hydroxy ethyl methacrylate, increases the color stability of these teeth.^8,17^

One of the limitations of the present study was the protocol used to evaluate color changes of the artificial teeth. Clinically, saliva prevents the continuous contact of solutions and drinks with the teeth. In addition, acrylic resin teeth are continuously cleaned with the use of mechanical methods, such as brushing, and chemical methods, such as mouthwashes, which can result in changes in the surface topography of these teeth, increasing their stability. In the present study, the solutions were evaluated at 37ºC. However, different drinks are used at different temperatures. In this context, tea and coffee are usually served hot, and soft drinks and different fruit juices are served cold. These temperature changes might affect the color stability of the teeth.^6^

Therefore, it is suggested that in future studies, the combined effects of different drinks and mechanical and chemical cleaning techniques of dentures and artificial teeth be evaluated.

## Conclusion

Different beverages gave rise to different types of color changes in different brands of acrylic resin teeth. The Ivoclar brand exhibited less color changes than the BStar and BetaDent brands. In the first week, all the three brands and in 30 days, the Ivoclar and BStar brands exhibited clinically acceptable color changes.

## Authors’ contributions

RM was the supervisor and designed the study. ZEA contributed to writing the manuscript and English editing. SMT contributed to the publication of the article and developing the protocol. FM contributed to the development of the protocol. NY contributed to the thesis, article writing, publication and data analysis. All the authors have read and agreed to the published version of the manuscript.

## Acknowledgments

The authors would like to thank the faculty of dentistry of Tabriz University of medical sciences for assistance provided during the experiments of this study

## Funding

This research was done without any financial support of institutes, factory, etc.

## Competing interests

The authors declare no competing interests with regards to the authorship and/or publication of this article.

## Ethics approval

The study protocol was approved by the Ethics Committee of Tabriz University of Medical Sciences (code TBZMED.VCR.REC.1397.249).
